# Supplementary material for: Success of community approach to HPV vaccination in school-based and non-school-based settings in Haiti
Source: PLoS One. 2021 Jun 24;16(6):e0252310. doi: 10.1371/journal.pone.0252310 (PMC8224934; doi:10.1371/journal.pone.0252310)
Supplement: S1 File — (PDF) [file pone.0252310.s001.pdf]

KesLJonèpoulaksinentifiσбƬσχlanekontkansèkòlmatris

PllojèVaksenHPVƬVaksinasLJonnanSantGHE

SKIO

No. kesyonè a : GHEƬ|___|___|___|__| - |_____| |___|__|

Dat : |___|___| |___|___|___| |___|
jou mwa lane

PATNETID : σƬ|___|___|___|__|___|___|

klinik : ____________________________

Ida ntif ik a s LJon r e s kons a b tim oun na n ;P a le a l è k r e kons a b tim oun na nJ

Nimewo : |___|___|___|__| Siyati : ________________________ Non : ________________________

Sèks : M ☐ F ☐ Laj : |___|___|

Relasyon ak timoun nan : ☐ Manman/Papa ☐ Gran sè/Gran frè ☐ Granmè/Granpè
☐ Matant/Mononk ☐ Gran kouzin/Gran kouzen ☐ Lòt relasyon : ________________________

Telefòn : |___|___|___|__|___|___|___|___| |___|___|___|__|___|___|__|___|

Adrès : ____

Q1 - Eske jèn fi ou reskonsab la pran tout dòz vaksen li sipoze pran yo deja? ☐ Wi ☐ Non

Q2 - Apre tout sa nou rakonte w sou kansè kòl matris, eske ou dakò pou jèn fi sa a ke ou reskonsab la pran vaksen pou pwoteje l kont maladi sa a? ☐ Wi ☐ Non

Q2A - Si wi, eske timoun nan ap disponib nan 6 mwa, pou li tounen sant GHESKIO pran dezyèm dòz la? ☐ Wi ☐ Non

Q2B - Si ou pa dakò, di pou ki rezon : ☐ Kwayans/Relijyon ☐ Mwen pa okouran de vaksen sa a
☐ Mwen pè pou piki a pa fèl malad ☐ Maladi sa a pa pou timoun mwen
☐ Lòt rezon : ____________________________________

Ida ntif ik a s LJon tim oun na n ;T i moun na n s e LJon ti fi̦ pa le a l èk liJ

Lèt alfabè : |______| Siyati : ________________________ Non : ________________________

Laj : |___|___|
|___|

Dat li fèt : |___|___| |___|__|___| |___|___|
jou mwa lane

Telefòn : |___|___|___|__|___|___|___|___| |___|___|___|__|___|___|__|___| Q3 - Eske ou swiv GHESKIO? ☐ Wi ☐ Non

Q4 - Eske ou janm ale lekòl? ☐ Wi ☐ Non Q4A - Si wi, ki dènye ane ke ou te ale? |___|___|___|
|

Q5 - Apre tout sa nou rakonte w sou kansè kòl matris, eske ou dakò pou ou pran vaksen pou pwoteje w kont maladi sa a? ☐ Wi ☐ Non

Q5A - Sinon, pou ki rezon? ☐ Kwayans/Relijyon ☐ Maladi sa a pa pou mwen ☐ Mwen pè piki
☐ Lòt rezon : ____________________________________

Is tll a sa nte tim oun na n a l a n pr e mLJe dònj l ak se n HP V a ;P a le a l èk r e kons a b tim oun na nJ

Q6 - Eske timoun nan ap swiv doktè pou pwoblèm sante oswa pou nenpòt ki lòt rezon kounyen a? ☐ Wi ☐ Non

Q6A - Si wi, pou ki rezon?____________________________________

Q7 - Eske timoun nan ap pran medikaman kounyen a? ☐ Wi ☐ Non

Q7A - Si wi, ki medikaman? ____________________________________

Q8 - Eske timoun nan gen bagay lè li manje oswa bwè oswa sèvi ak yo, yo leve sou li? ☐ Wi ☐ Non

Q8A - Si wi, kisa yo ye? ☐ Medikaman :____________________________
☐ Manje :____________________________
☐ Lòt bagay :____________________________

Eta s a nte tim oun na n a l an pr e mLJe dònj l a ks e n HP V a ;P a le a l èk tim oun na nJ

Qϵ - Eske ou fòme? ☐ Wi ☐ Non QϵA - Si wi, eske ou ansent? ☐ Wi ☐ Non

QϵB - Ki dat dènye fwa règ ou te vini? |___|___| |___|___|___| |___|__|___|
|
jou mwa lane

Q10 - Eske timoun nan malad grav nan pwen pou li ta wè doktè kounyen a? ☐ Wi ☐ Non

Q10A - Si wi, kisa li genyen? ☐ Lafyèv ☐ Souf kout ☐ Li touse anpil
☐ Vomisman ☐ Dyare ☐ Li pa ka kenbe kò l

☐ Lòt bagay :____

Q11 - Verifye ankò si timoun nan respekte tout kondisyon pou li pran vaksen an : ☐ Se yon fi

☐ Li genyen 10 - 14 lane ☐ Li pa ansent ☐ Li pa malad grav ☐ Lap disponib pou dezyèm dòz la

Q12 - Eske timoun nan elijib pou premye dòz vaksen HPV a? ☐ Wi ☐ Non

P r e mLJe dònj l a ks e n HP V

Q13 - Eske li pran vaksen an? ☐ Wi ☐ Non

Q13A - Si wi, nan ki ponyèt? ☐ Dwat ☐ Gòch

Q13B - Si non, pou ki rezon? ☐ Timoun nan chanje lide ☐ Reskonsab timoun nan chanje lide

☐ Lòt rezon : ____________________

Q14 - Eske timoun nan te gen konplikasyon nan 15 minit apre ou fin ba l vaksen an? ☐ Wi ☐ Non

Q14A - Si wi, eske eta li te nesesite yo ale avèk li Sant GHESKIO an ijans? ☐ Wi ☐ Non

Dat randevou pou dezyèm dòz la : |___|___| |___|___|___| |___|___| |___|

jou mwa lane

Siyati ak non vaksinatè a : ____________________ Ekip : |___|___| Operatè : ____________________

El aliLJ a sLJon pou e fe se gondè a pr e pr e mLJe dònj l a ks e n HP V a ;P a le a l èk tim oun na nJ

Dat evaliyasyon : |___|___| |___|___|___| |___|___| |__|

jou mwa lane

Q15 - Nan 2 jou ki vin apre premye dòz la eske ou te malad oswa ou te gen kèk malèz? ☐ Wi ☐ Non

Q15A - Si wi, kisa ou te genyen? ☐ Bra fè mal ☐ Tèt fè mal ☐ Tèt vire ☐ Pèt konesans ☐ Lafyèv ☐ Dyare ☐ Kè plen ☐ Vomisman ☐ Vant fè mal

☐ Lòt bagay : ____________________

Siyati ak non evaliyatè a : ____________________ Ekip : |___|___| Operatè : ____________________

Is tll a sa nte tim oun na n a l a n de njLJè m dònj l a ks e n HP V a ;P a le a l è k r e konsa b tim oun na nJ

Q16 - Eske timoun nan ap swiv doktè pou pwoblèm sante oswa pou nenpòt ki lòt rezon kounyen a? ☐ Wi ☐ Non

Q16A - Si wi, pou ki rezon? ____________________

Q17 - Eske timoun nan ap pran medikaman kounyen a? ☐ Wi ☐ Non

Q17A - Si wi, ki medikaman? ____________________

Q18 - Eske timoun nan gen bagay lè li manje oswa bwè oswa sèvi ak yo, yo leve sou li? ☐ Wi ☐ Non

Q18A - Si wi, kisa yo ye? ☐ Medikaman : ____________________

☐ Manje : ____________________

☐ Lòt bagay : ____________________

Eta s a nte tim oun na n a l an de njLJè m dònj l a ks e n HP V a ;P a le a l è k tim oun na nJ

Q1є - Eske ou fòme? ☐ Wi ☐ Non

Q1єA - Si wi, eske ou ansent? ☐ Wi ☐ Non

Q1єB - Ki dat dènye fwa règ ou te vini? |___|___| |___|___|___| |___|__|___| |

jou mwa lane

Q20 - Eske timoun nan malad grav nan pwen pou li ta wè doktè kounyen a? ☐ Wi ☐ Non

Q20A - Si wi, kisa li genyen? ☐ Lafyèv ☐ Souf kout ☐ Li touse anpil ☐ Vomisman ☐ Dyare ☐ Li pa ka kenbe kò l

☐ Lòt bagay : ____________________

Q21 - Verifye ankò si timoun nan respekte tout kondisyon pou li pran vaksen an :

☐ Li pran premye dòz la ☐ Li pa ansent ☐ Li pa malad grav

Q22 - Eske timoun nan elijib pou dezyèm dòz vaksen HPV a? ☐ Wi ☐ Non

D e njLJè m dònj l a ks e n HP V

Q23 - Eske li pran vaksen an? ☐ Wi ☐ Non

Q23A - Si wi, nan ki ponyèt? ☐ Dwat ☐ Gòch

Q23B - Si non, pou ki rezon? ☐ Timoun nan chanje lide ☐ Reskonsab timoun nan chanje lide

☐ Lòt rezon : ____________________

Q24 - Eske timoun nan te gen konplikasyon nan 15 minit apre ou fin ba l vaksen an? ☐ Wi ☐ Non

Q24A - Si wi, eske eta li te nesesite yo ale avèk li Sant GHESKIO an ijans? ☐ Wi ☐ Non

Siyati ak non vaksinatè a : ____________________ Ekip : |___|___| Operatè : ____________________

El aliLJ a sLJon pou e fe se gondè a pr e de njLJèm dònj l aks e n HP V a ;P a le a l è k tim oun na nJ

Dat evaliyasyon : |___|___| |___|___|___| |__|___| |__|

jou mwa lane

Q25 - Nan 2 jou ki vin apre dezyèm dòz la eske ou te malad oswa ou te gen kèk malèz? ☐ Wi ☐ Non

Q25A - Si wi, kisa ou te genyen? ☐ Bra fè mal ☐ Tèt fè mal ☐ Tèt vire ☐ Pèt konesans
☐ Lafyèv ☐ Dyare ☐ Kè plen ☐ Vomisman ☐ Vant fè mal

☐ Lòt bagay :____

Siyati ak non evaliyatè a :______________________________ Ekip : |___|___| Operatè :____________________

Kesyonè pou vaksinen ti fi 10 - 14 lane kont kansè kòl matris

Pwojè Vaksen HPV - Vaksinasyon a domisil

No. kesyonè a :

Dat : |__|__| |__|__|__| |__|__|__|__|
jou mwa lane

☐ Village de Dieu ☐ Cité Plus ☐ Cité l'Eternel ☐ Martissant Bolosse ☐ Carrefour-Feuille ☐ PAP Centre-Ville ☐

Blòk : |__|__| Adrès : ____________________

Idantifikasyon reskonsab timoun nan (Pale avèk rekonsab timoun nan)

Siyati : ____________________ Non : ____________________

Sèks : M ☐ F ☐ Laj : |__|__|

Relasyon ak timoun nan : ☐ Manman/Papa ☐ Gran sè/Gran frè ☐ Granmè/Granpè

☐ Matant/Mononk ☐ Gran kouzin/Gran kouzen ☐ Lòt relasyon : ____________________

Telefòn : |__|__|__|__|__|__|__|__| |__|__|__|__|__|__|__|__|

Q1 - Mande epi verifye kanè vaksinasyon timoun nan : ☐ Li ajou ☐ Li pa ajou ☐ Li pa disponib

Q1A - Si kanè vaksinasyon an pa disponib, eske timoun nan pran tout dòz vaksen li sipoze pran yo deja? ☐ Wi ☐ Non

Q2 - Apre tout sa nou rakonte w sou kansè kòl matris, eske ou dakò pou jèn fi sa a ke ou reskonsab la pran vaksen pou pwoteje l kont maladi sa a? ☐ Wi ☐ Non

Q2A - Si wi, eske timoun nan ap disponib nan 6 mwa, nan menm adrès sa a, pou li pran dezyèm dòz la? ☐ Wi ☐ Non

Q2B - Si ou pa dakò, di pou ki rezon : ☐ Kwayans/Relijyon ☐ Mwen pa okouran de vaksen sa a ☐ Mwen pè pou piki a pa fèl malad ☐ Maladi sa a pa pou timoun mwen ☐ Lòt rezon : ____________________

Idantifikasyon timoun nan (Timoun nan se yon ti fi, pale avèk li)

Siyati : ____________________ Non : ____________________

Laj : |__|__| Dat li fèt : |__|__| |__|__|__| |__|__|__|__|
jou mwa lane

Telefòn : |__|__|__|__|__|__|__|__| |__|__|__|__|__|__|__|__| Q3 - Eske ou swiv GHESKIO? ☐ Wi ☐ Non

Q4 - Eske ou janm ale lekòl? ☐ Wi ☐ Non Q4A - Si wi, ki dènye ane ke ou te ale? |__|__|__|__|

Q5 - Apre tout sa nou rakonte w sou kansè kòl matris, eske ou dakò pou ou pran vaksen pou pwoteje w kont maladi sa a? ☐ Wi ☐ Non

Q5A - Sinon, pou ki rezon? ☐ Kwayans/Relijyon ☐ Maladi sa a pa pou mwen ☐ Mwen pè piki ☐ Lòt rezon : ____________________

Istwa sante timoun nan avan premye dòz vaksen HPV a (Pale avèk rekonsab timoun nan)

Q6 - Eske timoun nan ap swiv doktè pou pwoblèm sante oswa pou nenpòt ki lòt rezon kounyen a? ☐ Wi ☐ Non

Q6A - Si wi, pou ki rezon?____________________

Q7 - Eske timoun nan ap pran medikaman kounyen a? ☐ Wi ☐ Non

Q7A - Si wi, ki medikaman? ____________________

Q8 - Eske timoun nan gen bagay lè li manje oswa bwè oswa sèvi ak yo, yo leve sou li? ☐ Wi ☐ Non

Q8A - Si wi, kisa yo ye? ☐ Medikaman :____________________ ☐ Manje :____________________ ☐ Lòt bagay :____________________

Eta sante timoun nan avan premye dòz vaksen HPV a (Pale avèk timoun nan)

Q9 - Eske ou fòme deja? ☐ Wi ☐ Non Q9A - Si wi, eske ou ansent? ☐ Wi ☐ Non

Q9B - Ki dat dènye fwa règ ou te vini? |__|__| |__|__|__| |__|__|__|__|
jou mwa lane

Q10 - Eske timoun nan malad grav nan pwen pou li ta wè doktè kounyen a? ☐ Wi ☐ Non

Q10A - Si wi, kisa li genyen? ☐ Lafyèv ☐ Souf kout ☐ Li touse anpil ☐ Vomisman ☐ Dyare ☐ Li pa ka kenbe kò l ☐ Lòt bagay :____________________

Q10C - Eske ou refere timoun nan bay Sant GHESKIO pou li konplete evaliyasyon an? ☐ Wi ☐ Non

Siyati ak non evaliyatè a :____________________ Ekip : |__|__| Operatè :____________________

Premye dòz vaksen HPV

Q11 - Verifye ankò si timoun nan respekte tout kondisyon pou li pran vaksen an : ☐ Se yon fi ☐ Li genyen 10 - 14 lane ☐ Li pa ansent ☐ Li pa malad grav ☐ Lap disponib pou dezyèm dòz la

Q12 - Eske timoun nan elijib pou premye dòz vaksen HPV a? ☐ Wi ☐ Non

Premye dòz vaksen HPV (swit)

Q13 - Eske li pran vaksen an? ☐ Wi ☐ Non

Q13A - Si wi, nan ki bra? ☐ Dwat ☐ Gòch

Nimewo vaksinasyon : **VHPV -** |__|__|__|__|

Dat premye dòz la : |__|__| jou |__|__|__| mwa |__|__|__|__| lane

Q13B - Si non, pou ki rezon? ☐ Timoun nan chanje lide ☐ Reskonsab timoun nan chanje lide

☐ Lòt rezon :____

Q14 - Eske timoun nan te gen konplikasyon nan 15 minit apre ou fin ba l vaksen an? ☐ Wi ☐ Non

Q14A - Si wi, li te nesesè yo ale avèk li Sant GHESKIO an ijans? ☐ Wi ☐ Non

Dat randevou pou dezyèm dòz la : |__|__| jou |__|__|__| mwa |__|__|__|__| lane

Siyati ak non vaksinatè a :_______________________ Ekip : |__|__| Operatè :_______________

Evaliyasyon pou efe segondè apre premye dòz vaksen HPV a (Pale avèk timoun nan)

Dat evaliyasyon : |__|__| jou |__|__|__| mwa |__|__|__|__| lane

Q15 - Nan 2 jou ki vin apre premye dòz la eske ou te malad oswa ou te gen kèk malèz? ☐ Wi ☐ Non

Q15A - Si wi, kisa ou te genyen? ☐ Bra fè mal ☐ Tèt fè mal ☐ Tèt vire ☐ Pèt konesans ☐ Lafyèv ☐ Dyare ☐ Kè plen ☐ Vomisman ☐ Vant fè mal

☐ Lòt bagay :____

Siyati ak non evaliyatè a :_______________________ Ekip : |__|__| Operatè :_______________

Istwa sante timoun nan avan dezyèm dòz vaksen HPV a (Pale avèk rekonsab timoun nan)

Dat evaliyasyon an : |__|__| jou |__|__|__| mwa |__|__|__|__| lane

Q16 - Eske timoun nan ap swiv doktè pou pwoblèm sante oswa pou nenpòt ki lòt rezon kounyen a? ☐ Wi ☐ Non

Q16A - Si wi, pou ki rezon?____

Q17 - Eske timoun nan ap pran medikaman kounyen a? ☐ Wi ☐ Non

Q17A - Si wi, ki medikaman? ____

Q18 - Eske timoun nan gen bagay lè li manje oswa bwè oswa sèvi ak yo, yo leve sou li? ☐ Wi ☐ Non

Q18A - Si wi, kisa yo ye? ☐ Medikaman :____

☐ Manje :____

☐ Lòt bagay :____

Eta sante timoun nan avan dezyèm dòz vaksen HPV a (Pale avèk timoun nan)

Q19 - Eske ou fòme deja? ☐ Wi ☐ Non

Q19A - Si wi, eske ou ansent? ☐ Wi ☐ Non

Q19B - Ki dat dènye fwa règ ou te vini? |__|__| jou |__|__|__| mwa |__|__|__|__| lane

Q20 - Eske timoun nan malad grav nan pwen pou li ta wè doktè kounyen a? ☐ Wi ☐ Non

Q20A - Si wi, kisa li genyen? ☐ Lafyèv ☐ Souf kout ☐ Li touse anpil ☐ Vomisman ☐ Dyare ☐ Li pa ka kenbe kò l

☐ Lòt bagay :____

Q20C - Eske ou refere timoun nan bay Sant GHESKIO pou li konplete evaliyasyon an? ☐ Wi ☐ Non

Siyati ak non evaliyatè a :_______________________ Ekip : |__|__| Operatè :_______________

Dezyèm dòz vaksen HPV

Q21 - Verifye ankò si timoun nan respekte tout kondisyon pou li pran vaksen an :

☐ Li pran premye dòz la ☐ Li pa ansent ☐ Li pa malad grav

Q22 - Eske timoun nan elijib pou dezyèm dòz vaksen HPV a? ☐ Wi ☐ Non

Q23 - Eske li pran vaksen an? ☐ Wi ☐ Non

Q23A - Si wi, nan ki bra? ☐ Dwat ☐ Gòch

Dat dezyèm dòz la : |__|__| jou |__|__|__| mwa |__|__|__|__| lane

Q23B - Si non, pou ki rezon? ☐ Timoun nan chanje lide ☐ Reskonsab timoun nan chanje lide

☐ Lòt rezon :____

Q24 - Eske timoun nan te gen konplikasyon nan 15 minit apre ou fin ba l vaksen an? ☐ Wi ☐ Non

Q24A - Si wi, eske li te nesesè yo ale avèk li Sant GHESKIO an ijans? ☐ Wi ☐ Non

Siyati ak non vaksinatè a :_______________________ Ekip : |__|__| Operatè :_______________

Evaliyasyon pou efe segondè apre dezyèm dòz vaksen HPV a (Pale avèk timoun nan)

Dat evaliyasyon : |__|__| jou |__|__|__| mwa |__|__|__|__| lane

Q25 - Nan 2 jou ki vin apre dezyèm dòz la eske ou te malad oswa ou te gen kèk malèz? ☐ Wi ☐ Non

Q25A - Si wi, kisa ou te genyen? ☐ Bra fè mal ☐ Tèt fè mal ☐ Tèt vire ☐ Pèt konesans ☐ Lafyèv ☐ Dyare ☐ Kè plen ☐ Vomisman ☐ Vant fè mal

☐ Lòt bagay :____

Siyati ak non evaliyatè a :____ Ekip : |__|__| Operatè :______________________
