# Supplementary material for: Success of community approach to HPV vaccination in school-based and non-school-based settings in Haiti
Source: PLoS One. 2021 Jun 24;16(6):e0252310. doi: 10.1371/journal.pone.0252310 (PMC8224934; doi:10.1371/journal.pone.0252310)
Supplement: Attachment — Submitted filename: Response to Reviewers_HPV-vaccination-study_02.15.2021.docx [file pone.0252310.s003.docx]

**Editor’s Comments**

This is a relevant work for Haiti and potentially for other Caribbean countries. This is a short report for a short community HPV vaccination campaign in Urban Haiti for adolescent girls.
It had a high degree of success given the rates they managed to reach.

**Comment 1:** For descriptive analysis on table 1, please add more descriptives for age of the guardian. Quartiles and range would be useful.

**Response to Comment 1:** We thank the Editor for this comment and have added the following information to Table 1, Pg. 10 for guardian age: minimum, maximum, 1^st^ quartile, median, 3^rd^ quartile.

**Comment 2:** Explain how the distance were measured. How this 1km cut-off was established.

**Response to Comment 2:** The 1km cut-off was selected *a priori* to data analysis as a proxy for whether participants had ease of access to a GHESKIO health facility. Distance was measured using the Google Maps ruler tool from GHESKIO clinic to the central point of each neighborhood. This information has also been added to the manuscript on Pg. 8:

“Logistic regressions were used to identify characteristics associated with HPV vaccination series completion; covariates included age (continuous), education level (lower than secondary school, secondary school), menarche (yes, no), having been previously followed as patient in the GHESKIO clinic (yes, no), neighborhood distance from GHESKIO clinic (<1 kilometer, ≥1 kilometer; 1 kilometer was selected as a proxy for whether participants had ease of access to a GHESKIO health facility, measured from GHESKIO clinic to the central point of each neighborhood using the Google Maps ruler tool), guardian age (continuous), and guardian relationship to participant (mother, other).”

**Comment 3:** How the adverse effects were collected. I do not see a description of this in the manuscript.

**Response to Comment 3:** We thank the Editor for their comment and agree that clarity with regards to how adverse effects were collected is needed. Adverse effects were collected within 15 minutes of each vaccination dose and recorded on that participants questionnaire. This information has also been added to the manuscript on Pg. 8:

“Side effects that occurred within 15 minutes of any vaccination does were recorded in the questionnaire.”

**Comment 4:** For the logistic regression: Good that all covariates were introduced. Is there any reason besides availability for these small set of covariates? Why the age of guardian in the regression was introduced as linear? Did you try more levels of distance?

**Response to Comment 4:** We thank the Editor for these suggestions. Covariates included in this analysis were constrained by data availability.

In response to the feedback on guardian age, we modeled guardian age as a categorical variable with four levels: 18 to 25 years, 26 to 35 years, 36 to 45 years, 46 or older. Girls with guardians aged 26 to 35 years had statistically significantly lower odds of receiving at least 1 dose. We have updated Table 2 to present the results of the regression using categorical instead of continuous guardian age as covariate (see Pg. 14 of the manuscript).

We did not initially attempt to stratify by additional levels of distance. A sensitivity analysis was performed to include 3 levels of distance: <1 km, 1 to 5 km, and >5 km from GHESKIO clinic. Odds ratios and significance of covariates are largely consistent with the original analysis, and no statistically significant difference between the three distance levels was observed. Hence, we did not change our analysis to add additional levels of distance, and have presented the sensitivity findings below:

**Table.** Multivariable Regression of Completion of the Vaccination Series Among Participating Adolescents Who Received at Least 1 Dose of HPV Vaccination, for stratified levels of distance

|  | | All participants (N=1,960) | | | |
| --- | --- | --- | --- | --- | --- |
| Variable | | **Adjusted OR** | **95% CI** | | ***P*-Value** |
| *Participant characteristics* | |  |  |  |  |
|  | Participant age (years) | 0.92 | (0.78, | 1.08) | 0.323 |
|  | Participant education |  |  |  |  |
|  | Secondary | 1.11 | (0.62, | 2.00) | 0.718 |
|  | <Secondary | Reference | | | |
|  | Experienced menarche |  |  |  |  |
|  | Yes | 1.87 | (1.11, | 3.14) | 0.018* |
|  | No | Reference | | | |
|  | GHESKIO patient |  |  |  |  |
|  | Yes | 2.26 | (1.36, | 3.75) | 0.002* |
|  | No | Reference | | | |
|  | Cohort |  |  |  |  |
|  | School | 3.96 | (2.06, | 7.61) | <0.001* |
|  | Non-school | Reference | | | |
|  | Neighborhood distance from GHESKIO clinic |  |  |  |  |
|  | <1km | 0.62 | (0.27, | 1.41) | 0.114 |
|  | 1km to 5km | 1.12 | (0.60, | 2.10) | 0.146 |
|  | >5km | Reference | | | |
| *Guardian characteristics* | |  |  |  |  |
|  | Guardian age (years) |  |  |  |  |
|  | 18 to 25 | 1.18 | (0.61, | 2.28) | 0.293 |
|  | 26 to 35 | 0.64 | (0.36, | 1.15) | 0.007* |
|  | 36 to 45 | 1.15 | (0.60, | 2.20) | 0.357 |
|  | 46 or older | Reference | | | |
|  | Guardian relationship |  | | | |
|  | Mother | 1.51 | (0.93, | 2.44) | 0.093 |
|  | Other | Reference | | | |

**Comment 5:** In a supplementary (not necessary for the main manuscript) would be good to have a more detailed list the guardian relationship

**Response to Comment 5:** The complete list of guardian relationships in the sample are as follows:

| Relationship | Count (%) |
| --- | --- |
| Mother/Father (“Manman/Papa”) | 1,162 (58.3%) |
| Neighbor (“Vwazin/Vwazine”) | 310 (15.6%) |
| Aunt/Uncle (“Matant/Mononk”) | 163 (8.2%) |
| Cousin (“Gran konzin/Gran kouzen”) | 152 (7.6%) |
| Older sister/Older brother (“Gran sè/Gran frè”) | 104 (5.2%) |
| Grandmother/Grandfather (“Granmè/Granpè”) | 34 (1.7%) |
| Godmother/Godfather (“Marenn/Parenn”) | 16 (0.8%) |
| Mother-in-law/father-in-law (“Bèl mè /Bo pè”) | 15 (0.8%) |
| Mother friend (“Zanmi manman”) | 14 (0.7%) |
| Other relation (“Lòt relasyon”), please specify | 24 (1.2%) |
| Caregiver (“Akonpayatè”) | 13 (0.7%) |
| Brother-in-law/Sister-in-law (“Bel sè / Bo frè”) | 4 (0.2%) |
| Godmother (“Makomè”) | 3 (0.2%) |
| Godson/Goddaughter (“Fiyel”) | 2 (0.1%) |
| Nephew/Niece (“Neve”) | 1 (0.1%) |
| Family (“Fanmi”) | 1 (0.1%) |

Detailed list of guardian relationships has been included in the manuscript as a footnote to Table 2, on Pg. 14.

**Reviewer #1 Comments**

In this paper the Authors performed an assessment of two modalities of delivering HPV vaccines to adolescent girls in an urban setting in Port-au-Prince Haiti. In particular, they estimated descriptive statistics and utilized logistic regression models to examine characteristics associated with completion of vaccination doses for each delivery method. This study is very interesting however 5 points need to be addressed:

**Comment 1:** Abstract page 2. Methods. Change this section to read “Data collection was conducted during HPV vaccination campaigns in Port -au -Prince between August 2016 and April 2017. Descriptive statistics and logistic regression models were used to examine characteristics associated with vaccination series completion of school based and non-school based vaccination delivery modalities.”

**Response to Comment 1:** We thank the reviewer for their comment and have incorporated the suggested language in the abstract on Pg. 2.

**Comment 2:** Abstract page 2. Conclusions. Rewrite to read "Vaccination in school- and non-school-based settings was successful, suggesting that a nationwide HPV vaccination campaign using either approach would be successful".

**Response to Comment 2:** See response to Comment 1.

**Comment 3:** Main text Methods and materials: Page 5 Line 77. End the sentence at after HPV vaccine. The next line 78 should then read “Awareness campaigns targeting adolescent girls were conducted at the GHESKIO clinic and throughout neighboring communities and target schools from July 2016 through April 2017”

**Response to Comment 3:** We have incorporated the suggested language on Pg. 5.

**Comment 4:** Literature review is not enough. There are some articles, which must be added to literature review: a) Soi, C. Human papillomavirus vaccine delivery in Mozambique: identification of implementation performance drivers using the Consolidated Framework for Implementation Research (CFIR); b) Soi, C. Implementation Strategy and Cost of Mozambique’s HPV vaccine demonstration project; c) Soi, C. Global health systems partnerships: A mixed methods analysis of HPV vaccine delivery network actors in Mozambique.

**Response to Comment 4:** The suggested references have been incorporated into the discussion section on Pg. 17:

“As previous research has shown, a successful expansion of an HPV vaccination campaign to a national-level in a low-to-middle-income country requires local and international support and financial investment [24-26]. A key aspect highlighted by similar programs in Mozambique was that out-of-school and unenrolled girls would be missed by school-based vaccination programs; an adaptable model that would allow communities to target this demographic via both school-based and non-school based vaccination programs is recommended. The high rate of successful completion in our study in both the non-school and school cohorts, provides evidence that such adaptable models would be appropriate in Haiti.”

**Comment 5:** DOI of all the references must be added (you can use "<https://crossref.org/>").

**Response to Comment 5:** DOI inputs for all references have been updated.

**Response to Journal Staff**

**Comment 1:** Please ensure that your manuscript meets PLOS ONE's style requirements, including those for file naming. The PLOS ONE style templates can be found at

https://journals.plos.org/plosone/s/file?id=wjVg/PLOSOne_formatting_sample_main_body.pdf and <https://journals.plos.org/plosone/s/file?id=ba62/PLOSOne_formatting_sample_title_authors_affiliations.pdf>

**Response to Comment 1:** The submission has been reformatted as requested.

**Comment 2:** Please provide additional details regarding participant consent. In the ethics statement in the Methods and online submission information, please ensure that you have specified what type you obtained (for instance, written or verbal, and if verbal, how it was documented and witnessed). If your study included minors, state whether you obtained consent from parents or guardians. If the need for consent was waived by the ethics committee, please include this information.

**Response to Comment 2:** Details regarding the nature of parental consent have been added to the manuscript in the Ethics Statement on Pg. 8-9:

This project was approved by the GHESKIO Institutional Review Board (Comite des Droits Humains) on June 25th, 2016. Parental consent was obtained prior to vaccination and documented in the study questionnaire; in the school cohort, written parental consent was obtained, and in the non-school cohort parental consent was obtained verbally.

Permission to conduct the project was obtained from the Ministry of Health (MSPP), who provided supervising staff to participate in this vaccination campaign.

**Comment 3:** During our internal checks, the in-house editorial staff noted that you conducted research or obtained samples in another country. Please check the relevant national regulations and laws applying to foreign researchers and state whether you obtained the required permits and approvals. Please address this in your ethics statement in both the manuscript and submission information.  In addition, please ensure that you have suitably acknowledged the contributions of any local collaborators involved in this work in your authorship list and/or Acknowledgements. Authorship criteria is based on the International Committee of Medical Journal Editors (ICMJE) Uniform Requirements for Manuscripts Submitted to Biomedical Journals - for further information please see here: https://journals.plos.org/plosone/s/authorship.

**Response to Comment 3:** Thank you for this comment. Our collaborators in Haiti have confirmed that the relevant national regulations and laws applying to foreign researchers have been met, and all required permits and approvals were obtained. We also confirm our acknowledgement of local collaborators, as well as included all contributors meeting ICMJE authorship criteria. The GHESKIO IRB approved this project on June 25th, 2016, and the Ministry of Health approved the project and provided a supervisor nurse to oversee vaccination activities. Please see updated language provided in Response to Comment 1.

**Comment 4** Thank you for stating the following in the Competing Interests section:

"The authors have declared that no competing interests exist." We note that one or more of the authors are employed by a commercial company: Analysis Group, Inc. Please provide an amended Funding Statement declaring this commercial affiliation, as well as a statement regarding the Role of Funders in your study. If the funding organization did not play a role in the study design, data collection and analysis, decision to publish, or preparation of the manuscript and only provided financial support in the form of authors' salaries and/or research materials, please review your statements relating to the author contributions, and ensure you have specifically and accurately indicated the role(s) that these authors had in your study. You can update author roles in the Author Contributions section of the online submission form.

Please also include the following statement within your amended Funding Statement.

“The funder provided support in the form of salaries for authors [insert relevant initials], but did not have any additional role in the study design, data collection and analysis, decision to publish, or preparation of the manuscript. The specific roles of these authors are articulated in the ‘author contributions’ section.”

If your commercial affiliation did play a role in your study, please state and explain this role within your updated Funding Statement.

Please also provide an updated Competing Interests Statement declaring this commercial affiliation along with any other relevant declarations relating to employment, consultancy, patents, products in development, or marketed products, etc.

Within your Competing Interests Statement, please confirm that this commercial affiliation does not alter your adherence to all PLOS ONE policies on sharing data and materials by including the following statement: "This does not alter our adherence to PLOS ONE policies on sharing data and materials.” (as detailed online in our guide for authors [http://journals.plos.org/plosone/s/competing-interests](about:blank)) . If this adherence statement is not accurate and  there are restrictions on sharing of data and/or materials, please state these. Please note that we cannot proceed with consideration of your article until this information has been declared.

Please include both an updated Funding Statement and Competing Interests Statement in your cover letter. We will change the online submission form on your behalf.

Please know it is PLOS ONE policy for corresponding authors to declare, on behalf of all authors, all potential competing interests for the purposes of transparency. PLOS defines a competing interest as anything that interferes with, or could reasonably be perceived as interfering with, the full and objective presentation, peer review, editorial decision-making, or publication of research or non-research articles submitted to one of the journals. Competing interests can be financial or non-financial, professional, or personal. Competing interests can arise in relationship to an organization or another person. Please follow this link to our website for more details on competing interests: [http://journals.plos.org/plosone/s/competing-interests](about:blank)

Response to Comment 4: We have updated our funding information (Pg. 20, also below). An updated competing interests statement is also included below. Both of these statements are reiterated in our cover letter as well.

“Funding: This project carried out at GHESKIO was supported by the MSPP, Partners in Health, and Merck Pharmaceuticals. No funding was received for the conduct of this project. The Analysis Group Inc. provided support in the form of salaries for authors [JC, SB, HC], but did not have any additional role in the study design, data collection and analysis, decision to publish, or preparation of the manuscript. The specific roles of these authors are articulated in the ‘Author Contributions’ section in the manuscript.”

“Competing Interests: Authors JS, SB, and HC were employees of Analysis Group Inc. at the time of manuscript development. This does not alter our adherence to PLOS ONE policies on sharing data and materials.”
