# Supplementary material for: Success of community approach to HPV vaccination in school-based and non-school-based settings in Haiti
Source: PLoS One. 2021 Jun 24;16(6):e0252310. doi: 10.1371/journal.pone.0252310 (PMC8224934; doi:10.1371/journal.pone.0252310)
Supplement: Attachment — Submitted filename: Response to Reviewers_HPV-vaccination-study_05.07.2021.docx [file pone.0252310.s004.docx]

**Reviewer #1 Comments**

**Comment 1**: I have some major concerns. I understood the study participants included patients with Kaposi sarcoma and healthy adolescent girls. This is not clear in the abstract , introduction and methodology.

Response 1: We thank the reviewer for this comment and the opportunity to make this clear in our manuscript. GHESKIO (the Haitian Study Group on Kaposi’s Sarcoma and Opportunistic Infections) is the community organization that conducted this study in Port-au-Prince. GHESKIO was founded in 1982 and was the world’s first institution dedicated to fighting HIV/AIDS. Today, GHESKIO provided HIV testing and treatment, maternal-child health and nutrition services, treatment for sexually transmitted infections, and many other social services.

In this study, the population included adolescent girls aged 9 to 14 who were willing to receive the vaccine and had parental consent (lines 97-98); the study population did not include individuals with Kaposi’s Sarcoma. In order to make this clear to readers, we have edited language in the manuscript from “GHESKIO patient” to “patient at a GHESKIO clinic” where applicable (Table 1, line 219, 222-223, 225, 228-230, 239, Table 2).

**Comment 2**: In the Line No 219-221 authors state that “Overall, the proportion of participants who were GHESKIO patients among those who completed the vaccination series was not quite statistically different from that of participants who have not been a patient at the clinic.” Article need more clarity.

Response 2: See Response 1. This statement refers to whether the participant was a former GHESKIO clinic patient or not. We have added a reference to Table 1 for clarity (lines 219-221).

**Comment 3**: Abstract Objectives Line No 24-26 Specify the age of study participants

Response 3: The abstract has been updated accordingly. See below (lines 24-26):

“Objectives. To assess the success of a human papillomavirus (HPV) vaccination program among adolescent girls aged 9-14 years in Haiti and to understand predictors of completion of a two-dose HPV vaccination series.”

**Comment 4:** Results Line 34 The term "Patient" is confusing, As I understood the study included both patients as well as healthy adolescents term patient to be replaced with vaccine recipients or vaccinees. Instead of patient history-medical/surgical history of study participants will be appropriate.

Response 4: See response 1. Additionally, we have updated the language to make clear that we are referring to whether the participant was a former patient at a GHESKIO clinic. See below (lines 34-35):

“Menarche (OR: 1.87; 95% CI, 1.11-3.14), if the participant was a prior patient at the GHESKIO clinics (OR: 2.17; 95% CI, 1.32-3.58), and participating in the school-based program (OR: 4.17; 95% CI, 2.14-8.12) were significantly associated with vaccination completion.”

**Comment 5:** Line No 72 GHESKIO -Is the vaccine introduced in only patients with Kaposi sarcoma. Mention other healthy participants.

Response 5: See response 1.

**Comment 6:** Line 81 Abbreviation for PIH

Response 6: We thank the reviewer for catching this. Lines 66-67 when the word is first used has been updated to include the acronym; see below:

“In 2009, a three-dose HPV vaccination pilot program was launched in Mirebalais, in central Haiti, supported by an initiative of Zanmi Lasante/Partners in Health (PIH).”

**Comment 7:** Line 102 Methods and materials, Define Patient /study participants

Response 7: See response 1.

**Comment 8:** Line 178 whereas only around one fifth (21.4%) were pregnant at the time of vaccination, all of whom received both doses of the vaccine. HPV vaccine is not administered in pregnant women. Justify this.

Response 8: We thank the reviewer for catching this. That number was an error resulting from our internal review process and was in reference to participants from neighborhoods within 1km of the GHESKIO clinic. See below (line 179-180):

“More than half (58.8%) of the non-school cohort were from neighborhoods within 1 kilometer of the GHESKIO clinic, whereas only around one fifth (21.4%) from neighborhoods within 1 kilometer of the GHESKIO clinic in the school cohort.“

**Comment 9**: Line 228 After adjusting for participant age, education, GHESKIO patient status-Authors have to define GHESKIO patient status

Response 9: See response 1. Additionally, we have updated the language to make clear that we are referring to whether the participant was a former patient at a GHESKIO clinic (lines 229-233). See below:

“After adjusting for participant age, education, whether the participant was a GHESKIO patient or not, cohort (school or non-school), neighborhood distance from GHESKIO clinic, and guardian characteristics, girls who had experienced menarche had 1.87 times higher odds of completing the vaccination series than those who had not experienced menarche (95% confidence interval [CI], 1.11-3.14; *P*=0.019; Table 2).”
