# Supplementary material for: Success of community approach to HPV vaccination in school-based and non-school-based settings in Haiti
Source: PLoS One. 2021 Jun 24;16(6):e0252310. doi: 10.1371/journal.pone.0252310 (PMC8224934; doi:10.1371/journal.pone.0252310)
Supplement: Attachment — Submitted filename: PONE-D-20-36531.docx [file pone.0252310.s005.docx]

Authors have addressed all the concerns.

Manuscript can be accepted.
